# Effect of Satureja *khuzestanica* essential oil against fertility disorders induced by busulfan in female mice

**Published:** 2017-12-15

**Authors:** Abbas Ahmadi, Saleh Bamohabat Chafjiri, Rajab Ali Sadrkhanlou

**Affiliations:** *Department of Basic Sciences, * *Faculty of Veterinary Medicine, Urmia University* *, Urmia, Iran.*

**Keywords:** Busulfan, Infertility, Mouse, Ovary, *Saturejakhuzestanica*

## Abstract

Busulfan is an alkylating agent affects ovarian follicles growth by oxidative stress induction. *Satureja khuzestanica* has antioxidant effects. The aim of this study was to examine whether *S. khuzestanica* essential oil (SKEO) exhibits protective effects on busulfan-induced ovarian failure. Eighty-four adult female mice were divided into six groups including dimethyl sulfoxide (control), SKEO 225.00 mg kg^-1^ (orally), busulfan 3.00 mg kg^-1^ (orally), busulfan 36.00 mg kg^-1^ (intraperitoneally), busulfan 3.00 mg kg^-1^ and SKEO and busulfan 36.00 mg kg^-1^ and SKEO. After 28 days, the mice were euthanized and oocytes were removed for *in vitro* fertilization (IVF) rate evaluation. Oocyte quantity and quality, fertilization rate and pre-implantation embryo development were daily examined with a stereo microscope in a period of 120 hr. Serum levels of estradiol and progesterone were also evaluated. Busulfan caused significant decreases in oocyte number and quality, fertilization rate, pre-implantation embryo development and embryo quality. The SKEO significantly decreased the adverse effects of busulfan. The present study indicated that SKEO can protect female fertility potential against busulfan induced damages.

## Introduction

Due to increasing the prevalence of cancer especially in women, as a result, the affected women are exposed to anti-cancer drugs, their reproductive system and fertility may be influenced by chemotherapy.^1^^-^^3^ Chemotherapy impairs reproductive functions^4^ and causes follicular damage that can be resulted in the failure of ovarian pre-maturation and maturation. Therefore, protection against infertility caused by chemotherapy is necessary.^1^ Busulfan is a cancer drug used to treat breast and ovarian cancers as well as chronic myeloid leukemia.^5^ It is a bifunctional alkylating agent producing reactive carbonium ions that can alkylate DNA and affect ovarian follicles growth. Alkylations of DNA within the nucleus probably represent the major interactions leading to cell death. However, these drugs react chemically with sulfhydryl, amino, hydroxyl, carboxyl and phosphate groups of other cellular nucleophiles.^5^ The alkylating agents- associated adverse effects are usually dose-related and occur primarily in rapidly growing tissues such as bone marrow, gastrointestinal tract and reproductive system.^5^ High-dose busulfan is a major cause of ovarian failure even when given in pre-pubertal period.^6 ^Busulfan can stimulate oxidative stress via glutathione (GSH) depletion and thioredoxin reductase inhibition.^7^^,^^8^ Busulfan decreases enzymatic antioxidants levels including glutathione peroxidase and superoxide dismutase and increases the level of lipid peroxidation in sperms and testicular tissues of treated mice. Increase of oxidative stress and high levels of lipid peroxidation are related to increased reactive oxygen species (ROS) generation in testes and epididymal sperms of busulfan treated animals.^9^ Thus, the antioxidant drugs including herbal agents can protect against cell and tissue damages induced by busulfan. 

One of the plants with potential anti-oxidant activity is *Satureja khuzestanica.* (Marzeh khuzestani in Persian, from Lamiaceae family), an endemic plant widely distributed in southern parts of Iran.^10^ In the folk medicine, this plant has been used as analgesic and antiseptic by inhabitants of southern parts of Iran. The chemical composition of essential oils of wild and cultivated *S. khuzestanica* have been determined.^11^ Flavonoids, pcymene, and carvacrol have been found as main constituents of the Iranian *S. khuzestanica*.^12^ Previous studies have shown concomitant antioxidant and antidiabetic effects of *S. khuzestanica* essential oil (SKEO) in streptozotocin-induced diabetic rats.^13 ^The antioxidant, antidiabetic, antihyperlipidemic, antifungal and antileishmanial activities of SKEO, and also its fertility and reproduction stimulating and anti-cholitis properties have been reported in experimental models.^12^^-^^18^ *Satureja khuzestanica* significantly decreases fasting blood glucose and triglyceride levels in diabetic and hyperlipidemic rats,^13^ and improves patients’ total cholesterol, low- and high-density lipoprotein cholesterol and total antioxidant power status.^19^ Previous human and animal studies have showed a significant antioxidative potential of SKEO.^20^

In this study, we used SKEO as a protective agent against the side effects of busulfan on ovarian follicles and female mice fertility. 

## Materials and Methods

Eighty-four adult female mice (8 weeks, 25.00 ± 2.00 g) were used in this study (10 mice for IVF and four mice for hormonal assay in each group). All animals were housed under standard conditions of temperature 22.00 ± 2.00 ˚C, 30.00 to 60.00 %, the light period of 14 hr and 10 hr of darkness and allowed to have free access to normal food and water. All experimental protocols were approved by the ethical committee of Urmia University based on proofed principles for laboratory animal care (P/3/1, 1390).

**Chemicals.** The aerial parts of *S. khuzestanica* plant were collected during the flowering stage of the plant from Khoaramabad (Lorestan province, Iran). The plant’s aerial parts were air dried at ambient temperature in the shade and hydro distilled using a clevenger type apparatus for 5 hr, giving yellow oil in 0.90% yield. The oils were dried over anhydrous sodium sulfate and stored at 4 ˚C. The density of the essence was 0.98.^21^ The SKEO was dissolved in distilled water and twin 20. Busulfan (Sigma, St. Louis, USA) was ﬁrst dissolved in dimethyl sulfoxide (DMSO; 10.00 mg mL^-1^). The same volume of distilled water was added just before use (ﬁnal concentration 5.0 mg mL^-1^).^22^

**Study design.** Animals were randomly divided into six groups, each group consists of ten animals and all groups treated for 28 days. Treatment groups were as follows: group 1 (control) received only vehicle (DMSO and distilled water) orally once a day; group 2 was gavaged with SKEO at the dose of 225.0 mg kg^-1^ once a day; group 3 was gavaged with busulfan solution at a dose of 3.00 mg kg^-1^ once a day; group 4 busulfan solution was given intraperitoneally at a dose of 36.00 mg kg^-1^; group 5 was gavaged with both busulfan solution at a dose of 3.00 mg kg^-1^ and SKEO at a dose of 225.00 mg kg^-1^ once a day and group 6 received both busulfan solution intraperitoneally at a dose of 36.00 mg kg^-1^ and SKEO orally at a dose of 225.00 mg kg^-1^ once a day.^9^^,^^23^^-^^25^

**Gonadotropins injections.** After 28 days, to induce superovulation in selected mice of each group, 10 IU pregnant mare’s serum gonadotrophin hormone (Intervet International BV, Boxmeer, The Netherlands) was injected intraperitoneally and after 48 hr intraperitoneal injection of 10 IU human chorionic gonadotrophin hormone (hCG) (Intervet International BV) was performed.^19^^,^^26^

**Preparation of IVF culture medium.** A day before fertilization, the necessary culture media for fertilization were prepared and then, incubated at 37 ˚C under 5% CO_2_ for 12 hr to reach stability. Fertilization dishes were dropped with human tubal fluid (HTF; Sigma, St. Louis, USA) medium. One droplet of 500 μL was placed in each dish for fertilization and several 100 μL droplets were located in dishes for elution. All droplets were covered with mineral oil. 

**Collecting of oocytes and IVF.** Approximately 13 hr after hCG injection (next morning), the female mice were euthanized. After shaving and sterilizing the abdominal area and laparotomy, the oviducts were detached and placed in culture medium stabilized at 37 ˚C. Then, the oocytes were removed using a dissecting method and added to fertilization droplets under mineral oil in 4 mg mL^-1^ HTF-bovine serum albumin (BSA) medium after washing. 

For sperm sampling, a male mouse was sacrificed 15 to 45 min before oocyte collection. Cauda epididymis was dissected out along with vas deferens. They were placed in the sperm dish and epididymis was minced by making 5-7 slashes with the needle of an insulin syringe and incubated at 37 ^°^C, 5% CO_2_, and the sperms were allowed to swim out for 15 min and counted. Then, motivated and capacitated sperms with the concentration of 1 × 10^6^ total sperm per ml of culture medium were added. 

Fertilization process was recognized 3 to 5 hr after adding sperms through observing two pronuclei. Therefore, fertilized oocytes (zygotes) were cultured in 100 mL droplets under mineral oil for 120 hr.^23^ About 24 hr after zygotes culture, the numbers of two-cell embryos were counted and after 120 hr, development of blastocysts, as well as arrested embryos were evaluated under invert microscopy (model IX70; Olympus, Tokyo, Japan).

Classification of arrested embryos according to their fragmentation and necrosis was as follow s: ^24^ Type I: Fully cellular lysing, necrotic and/or fragmented embryos; Type II: Embryos with partially fragmented blastomeres; Type III: Embryos with some fragmented blastomeres and/or cytoplasmic vesicles.^19^^,^^26^

The two-cell embryos were transferred from each wash dish to one of the drops of HTF medium with 4 mg mL^-1^ BSA from the corresponding culture dish. They were then washed three times and distributed to the rest of the drops (to remove the HTF) for another stages culture. The number of oocytes and oocytes quality, fertilization rate, and pre-implantation embryo development were daily examined with stereo microscope for five days.^19^^,^^26^

**Serological analyses. **Selected animals of each group were anesthetically euthanized 24 hr after the last treatment, and blood samples were collected from the animal’s heart. Then, the sera were separated by centrifuge at 1000 g for 15 min and stored at -70 ˚C until estradiol and progesterone assessments.

Serum concentrations of estradiol and progesterone were determined by competitive radioimmunoassay using commercial radioimmunoassay kits (Neogen, Tehran, Iran).

**Statistical** **analysis. **The IVF data were analyzed by Minitab (version 10.0; Minitab Inc., Boston, USA). Two proportion and hormones data were examined using one-way ANOVA followed by Tukey test using SPSS (version 16.0; SPSS Inc., Chicago, USA).

## Results

Data of oocytes quality, *in vitro* fertilization rate and embryonic development in different groups, are presented in Table 1. Percentages of proper oocyte and fertilization rates in busulfan-treated groups were significantly lower than the control group (*p* < 0.05). The percentage of fertilization was significantly increased by SKEO compared to control group. Furthermore, SKEO co-treatments in group 6 caused significant increase in IVF parameters in comparison with busulfan-treated groups (*p* < 0.05).

The percentages of two-cell embryos as an indicator of cleavage initiation showed significant decreases in busulfan-treated groups compared to the control group (*p* < 0.05). Percentages of two-cell embryos in busulfan and SKEO groups showed significant increases in comparison with busulfan-treated groups (*p* < 0.05).

Percentages of blastocysts in busulfan-treated groups showed significant decreases in comparison with the control group *(p* < 0.05). In SKEO group, the percentage of blastocyst increased significantly compared to the control group. The percentages of blastocysts in busulfan and SKEO groups showed significant increases compared to busulfan-treated groups and no significant difference in comparison with control group (*p* < 0.05). However, busulfan-treated groups showed significant differences with control group regarding this parameter (*p* < 0.05).

In busulfan-treated groups, the total percentages of arrested embryos in different developmental stages prior to blastocyst stage increased in comparison with the control group (*p* < 0.05). But, there were no significant differences between busulfan and SKEO groups with the control group, while other groups showed significant decreases compared to the control group (*p* < 0.05).

**Fig. 1:**
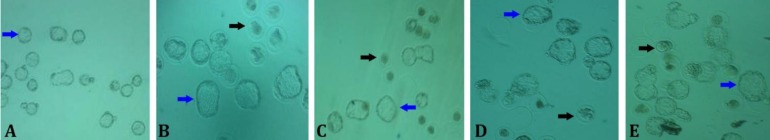
Representative photomicrographs of *in vitro* embryo development: A) Shows normal blastocysts in SKEO-treated group (100×); B) Busulfan 3.00 mg kg^-1^ group (200×); C) Busulfan 36.00 mg kg^-1^ group: Embryos arrested and type I, II and III of embryo quality (100×); D) Busulfan 3.00 mg kg^-1^ and SKEO group (100×); E) Busulfan 36.00 mg kg^-1^ and SKEO group (100×). Blue and black arrows indicate normal and arrested embryos, respectively.

Significant increases in the number of arrested embryos in busulfan-treated groups were observed compared to the control group (*p* < 0.05), while in busulfan and SKEO groups decreased the levels of fragmented and lysed embryos were observed in comparison with busulfan-treated groups (*p* < 0.05), (Table 1 and Fig. 1).

Serological data are shown in Table 2. Treatment by busulfan induced significant decreases in estradiol and progesterone levels compared to the control group. Hormone levels in SKEO and busulfan treated mice were increased significantly compared to the busulfan-treated groups (*p *< 0.05), (Table 2).

**Table 1:** Comparative assessment of oocyte quality and quantity, fertilization and embryo developmental rates in the control and experimental groups. Data are presented as number (%).

**Groups**	**Total number of oocytes**	**Proper oocytes**	**Fertilization rate**	**Two- Cell** **Embryos**	**Blastocyst**	**Arrested embryos**			
						**Total**	**Type I**	**Type ** **II**	**Type III**
**1 (Control)**	164	161(98.18)	153(95.04)	140(91.51)	100(65.36)	40(26.15)	3(1.97)	12(7.85)	25(17.65)
**2**	193	193(100.00)	193(100.00) ^a^	193(100.00)	176(91.20) ^a^	17(8.81)^a^	0(0.00)	6(3.11)	11(6.22) ^a^
**3**	338	280(82.85) ^a^ ^b^	230(82.15) ^a^ ^b^	170(73.92) ^a^ ^b^	142(61.74) ^a^ ^b^	88(37.27) ^a^ ^b^	30(13.05) ^a^ ^b^	26(11.31) ^a^ ^b^	32(13.92) ^a^ ^b^
**4**	108	80(74.08) ^a^ ^b^	44(55.00) ^a^ ^b^ ^c^	24(54.55) ^a^ ^b^ ^c^	4(9.10) ^a^ ^b^ ^c^	40(90.91) ^a^^ b ^^c^	8(18.19) ^a^ ^b^	20(45.46) ^a^ ^c^	12(27.28) ^a^ ^b^
**5**	150	130(86.67) ^a^ ^b^^ d^	115(88.47) ^b^ ^d^	111(96.53) ^c^ ^d^	70(60.87) ^b^ ^d^	45(39.14) ^a^^ b ^^d^	25(21.74) ^a^^ b ^^c^	11(9.57) ^d^ ^b^	9(7.83) ^a^^ d^
**6**	266	222(83.46) ^a^ ^b^ ^d^	176(79.28) ^a^ ^b^ ^d^ ^e^	138(78.41) ^a^ ^b^ ^d^	114(64.78) ^b^ ^d^	62(35.23) ^b^ ^d^	16(9.10) ^a^ ^b^ ^e^	20(11.37) ^d^ ^b^	26(14.78) ^b^

Different superscript letters indicate significant differences as follow:

a compared to control group,

b compared to 225.00 mg kg^-1^ SKEO (group 2),

c compared to 3.00 mg kg^-1^ busulfan (group 3),

d compared to 36.00 mg kg^-1^ busulfan (group 4),

e compared to 3.00 mg kg^-1^ busulfan and 225.00 mg kg^-1^ SKEO (group 6) at *p *< 0.05.

**Table 2:** Comparative assessment of serum concentrations of sex steroids in the control and experimental groups (mean ± SEM).

**Groups**	**Estradiol (pg mL** ^-1^ **)**	**Progesterone (ng mL** ^-1^ **)**
**1 (Control)**	41.80 ± 0.31	5.30 ± 0.05
**2**	64.70 ± 1.43^a^	8.07 ± 0.28 ^a^
**3**	21.70 ± 1.58 ^a^ ^b^	2.02 ± 0.26 ^a^ ^b^
**4**	27.80 ± 1.19 ^a^ ^b^	2.07 ± 0.17 ^a^ ^b^
**5**	34.30 ± 1.25 ^a^ ^b^ ^c^ ^d^	4.52 ± 0.08 ^b^ ^c^ ^d^
**6**	35.10 ± 1.05 ^b^ ^c^ ^d^	4.05 ± 0.09 ^b^ ^c^ ^d^

Different superscript letters indicate significant differences as follow:

a compared to control group,

b compared to 225.00 mg kg^-1^ SKEO (group 2),

c compared to 3.00 mg kg^-1^ busulfan (group 3),

d compared to 36.00 mg kg^-1^ busulfan (group 4),

## Discussion

Our findings showed that following busulfan treatment, percentages of the proper oocyte, fertilization rate, two cell embryo and blastocysts decreased significantly compared to the control group and percentage of arrested embryos increased significantly.

In a study, girls who had received busulfan all had evidence of ovarian failure based on lack of pubertal development and raised basal serum gonadotropin concentrations. High dose of busulfan is a major cause of ovarian failure in girls conditioned with high-dose of chemotherapy drugs, even during the pre-pubertal period.^6^ It has been reported that busulfan induces side effects in male reproductive system by loss of spermatogonia through apoptosis.^27^

Administration of a single dose of 20.00 mg kg^-1^ busulfan significantly increased the number of atrophied follicles, indicating adverse effects of busulfan on proliferating cells. Busulfan impacts negatively on cells with greater dividing abilities via DNA alkylation.^28^ In another report, busulfan/cyclophosphamide treatment which used as a single injection caused a gradual follicle loss and low estradiol and progesterone levels in treated mice.^29^ Busulfan treatment produced significant reductions in oocytes number and ovarian weight and volume when injected on day 12 of gestation.^30^ Administration of busulfan in pregnant rats has shown that effects of busulfan on fetal and neonatal rat ovaries depend on age at the time of treatment. The maximum side effect of busulfan resulted in the sterility of all female offspring, was observed following the treatment between the 13^th^ and 16^th^ days of gestation. The majority of offspring treated on day 11 of pregnancy and all those treated on day 18 were fully fertile.^31^

Comparison of *in* *vitro* fertilization potentials in SKEO-treated mice with the control group showed an increase in percentages of fertilization and blastocyst rates. We also found that in SKEO-received groups percentages of the proper oocyte, fertilization, two cell embryo and blastocyst were increased significantly compared to busulfan-treated groups. The percentage of arrested embryos decreased significantly following SKEO treatment compared to busulfan-treated groups (especially arrested embryos type II). Busulfan profoundly depleted GSH, confirming that its toxicity is mediated by oxidative stress induction and can be prevented by GSH level restoration.^32^

Previous findings confirmed the protective effects and usefulness of antioxidants in infertility and reproduction system disorders.^20^ Reportedly, human and animal studies have shown significant anti-oxidative potentials of *S. khuzestanica*.^32^ Oral administration of SKEO in male rats has resulted in significant improvements in all parameters of libido such as potency, fecundity, fertility index and litter size. Moreover, concentrations of FSH and testosterone and weights of testis, seminal vesicle and ventral prostate were significantly increased. Histo-pathological analyses also showed that numbers of spermatogonium, spermatid, Leydig cell and spermatozoid were increased and Sertoli cells were hypertrophic.^15^

Antioxidant effects of SKEO were reported by several researchers. Assaei *et al*. reported that hyperthyroidism induces marked hepatic toxicity through induction of oxidative toxic stress that can be prevented by SKEO and vitamin E. Furthermore, carvacrol as a major monoterpenic phenol of SKEO can inhibit arachidonic acid metabolism and ROS production leading to lipid peroxidation reduction.^33^ High antioxidant activity as well as remarkable antibacterial, analgesic, anti-inflammatory,^25^^,^^34^^,^^35^ and fertility and reproduction stimulating effects of SKEO ^12^^,^^13^ may play roles in busulfan-induced infertility prevention. Thus, pre-treatment and/or co-treatment with SEKO could decrease cytotoxic effects of busulfan on seminiferous tubules and cause sperm parameters improvement during chemotherapy.^9^

 In conclusion, SKEO may be effective against adverse effects of busulfan therapy in mice.

## References

[1] Blumenfeld Z (2012). Chemotherapy and fertility. Best Pract Res Clin Obstet Gynaecol.

[2] Georgescu ES, Goldberg JM, du Plessiset SS (2008). Present and future fertility preservation strategies for female cancer patients. Obstet Gynecol Surv.

[3] Jemal A, Bray F, Centeret M (2011). Global cancer statistics. CA Cancer J Clin.

[4] Madsen BL, Giudice L, Donaldson SS (1995). Radiation-induced premature menopause: A misconception. Int J Radiat Oncol Biol Phys.

[5] Katzung BG, Masters SB, Trevor AJ (2012). Basic and clinical pharmacology.

[6] Teinturier C, Hartmann O, Valteaucouanet D (1998). Ovarian function after autologous bone marrow transplantation in childhood: high-dose busulfan is a major cause of ovarian failure. Bone Marrow Transplant.

[7] Probin V, Wang Y, Bai A (2006). Busulfan selectively induces cellular senescence but not apoptosis in WI38 fibroblasts via a p53-independent but extracellular signal regulated kinase-p38 mitogen- activated protein kinase dependent mechanism. J Pharmacol Exp Ther.

[8] Probin V, Wang Y, Zhou D (2007). Busulfan-induced up senescence is dependent upon ROS production upstream of the MAPK pathway. Free Radic Biol Med.

[9] Nasimi P, Vahdati A, Tabandeh MR (2016). Cytoprotective and anti-apoptotic effects of Satureja khuzestanica essential oil against busulfan-mediated sperm damage and seminiferous tubules destruction in adult male mice. Andrologia.

[10] Jamzad Z (1994). New species of the genus Satureja (Labiatae) from Iran. Iran J Bot.

[11] Farsam H, Amanlou M, Radpour MR (2004). Composition of the essential oils of wild and cultivated Satureja khuzestanica Jamzad from Iran. Flavour Fragr J.

[12] Sefidkon F, Ahmadi S (2000). Essential oil of Satureja khuzestanica Jamzad. J Essent Oil Res.

[13] Abdollahi M, Salehnia A, Mortazavi SH (2003). Anti-oxidant, antidiabetic, antihyperlipidemic, reproduction stimulatory properties and safety of essential oil of Satureja khuzestanica in rat in vivo: A toxicopharmacological study. Med Sci Monit.

[14] Ghazanfari GH, Minaie B, Yasa N (2006). Biochemical and histopathological evidences for beneficial effects of Satureja khuzestanica Jamzad essential oil on the mouse model of inflammatory bowel diseases. Toxicol Mech and Methods.

[15] Haeri S, Minaie B, Amin GH (2006). Effect of Satureja khuzestanica essential oil on male rat fertility. Fitoterapia.

[16] Kheirandish F, Delfan B, Farhadi S (2011). The effect of Satureja khuzestanica essential oil on the lesions induced by Leishmania major in BALB/c mice. Afr J Pharm Pharmacol.

[17] Sadeghi-Nejad B, Saki J, Khademvatan S (2011). In vitro antileishmanial activity of the medicinal plant - Satureja khuzestanica Jamzad. J Med Plants Res.

[18] Sadeghi-Nejad B, Shiravi F, Ghanbari S (2010). Antifungal activity of Satureja khuzestanica (Jamzad) leaves extracts. Jundishapur J Microbiol.

[19] Zahmatkesh E, Najafi G, Nejati V (2015). Protective effect of royal jelly on in vitro fertilization (IVF) in male mice treated with oxymetholone. Cell J.

[20] Safarnavadeh T, Rastegarpanah M (2011). Antioxidants and infertility treatment, the role of Satureja Khuzestanica: A mini-systematic review. Iran J Reprod Med.

[21] Basiri S, Esmaily H, Vosough-Ghanbari S (2007). Improvement by Satureja khuzestanica essential oil of malathion-induced red blood cells acetylcholinesterase inhibition and altered hepatic mitochondrial glycogen phosphorylase and phosphoenolpyruvate carboxy-kinase activities. Pest Biochem Physiol.

[22] Udagawa K, Ogawa T, Watanabe T (2001). GnRH analog, leuprorelin acetate, promotes regeneration of rat spermatogenesis after severe chemical damage. Int J Urol.

[23] Ghasemi MF, Soleimani Rad J, Ghanbari AA (2008). An ultrastructural study on the apoptotic features of spermatogenic cells following busulfan treatment in adult mice. J Reprod Infertil.

[24] Ghasemi MF, Bahadori MH, Faghani M (2009). Buserelin inhibits apoptosis in male germ cells induced by busulfan in mouse testis. J Iran Anat Sci.

[25] Amanlou M, Dadkhah F, Salehnia A (2005). An anti-inflammatory and antinociceptive effects of hydro-alcoholic extract of Satureja khuzestanica Jamzad extract. J Pharm Pharm Sci.

[26] Murphy D, Carter DA (1993). Methods in molecular biology. Transgenesis techniques principles and protocols.

[27] Choi YJ, Ok DW, Kwon DN (2004). Murine male germ cell apoptosis induced by busulfan treatment correlates with loss of c-kit-expression in a Fas/FasL- and p53-independent manner. FEBS Letters.

[28] Pazhuhi H, Pousty I, Tajik P (2015). Protective effects of melatonin on mature ovarian follicles structures in adult mice treated with busulfan. Int J Biol Pharm Allied Sci.

[29] Jiang Y, Zhao J, Qi HJ (2013). Accelerated ovarian aging in mice by treatment of busulfan and cyclophosphamide. J Zhejiang Univ Sci B.

[30] Pelloux MC, Picon R, Gangnerau MN (1988). Effects of busulfan on ovarian folliculogenesis, steroidogenesis and anti-müllerian activity of rat neonates. Acta Endocrinol (Copenh).

[31] Hemsworth B N, Jackson H (1963). Effect of busulfan on the developing ovary in the rat. J Reprod Fertil.

[32] DeLeve LD, Wang XD (2000). Role of oxidative stress and glutathione in busulfan toxicity in cultured murine hepatocytes. Pharmacology.

[33] Assaei R, Zal F, Mostafavi-Pou Z (2014). Hepato-protective effect of Satureja Khuzestanica essential oil and vitamin E in experimental hyperthyroid rats: Evidence for role of antioxidant effect. Iran J Med Sci.

[34] Amanlou M, Fazeli MR, Arvin A (2004). Antimicrobial activity of crude methanolic extract of Satureja khuzestanica. Fitoterapia.

[35] Didry N, Dubrevil L, Pinkas M (1994). Activity of thymol, carvacrol, cinnamaldehyde and eugenol on oral bacteria. Pharm Acta Helv.

